# The Long-Term Effects of Prematurity and Intrauterine Growth Restriction on Cardiovascular, Renal, and Metabolic Function

**DOI:** 10.1155/2010/280402

**Published:** 2010-12-14

**Authors:** Patricia Y. L. Chan, Jonathan M. Morris, Garth I. Leslie, Patrick J. Kelly, Eileen D. M. Gallery

**Affiliations:** ^1^Perinatal Research, Kolling Institute of Medical Research, The University of Sydney, Royal North Shore Hospital, NSW 2065, Australia; ^2^Department of Obstetrics, Gynecology and Neonatology , Royal North Shore Hospital, NSW 2065, Australia; ^3^Neonatal Pediatrics, North Shore Private Hospital, Westbourne Street, St Leonards, NSW 2065, Australia; ^4^Biostatistics, Sydney School of Public Health, The University of Sydney, NSW 2006, Australia; ^5^Department of Renal Medicine, Royal North Shore Hospital, NSW 2065, Australia

## Abstract

*Objective*. To determine relative influences of intrauterine growth restriction (IUGR) and preterm birth on risks of cardiovascular, renal, or metabolic dysfunction in adolescent children. *Study Design*. Retrospective cohort study. 71 periadolescent children were classified into four groups: premature small for gestational age (SGA), premature appropriate for gestational age (AGA), term SGA, and term AGA. *Outcome Measures*. Systolic blood pressure (SBP), augmentation index (Al), glomerular filtration rate (GFR) following protein load; plasma glucose and serum insulin levels. *Results*. SGA had higher SBP (average 4.6 mmHg) and lower GFR following protein load (average 28.5 mL/min/1.73 m^2^) than AGA. There was no effect of prematurity on SBP (*P* = .4) or GFR (*P* = .9). Both prematurity and SGA were associated with higher AI (average 9.7%) and higher serum insulin levels 2 hr after glucose load (average 15.5 mIU/L) than all other groups. *Conclusion*. IUGR is a more significant risk factor than preterm birth for later systolic hypertension and renal dysfunction. Among children born preterm, those who are also SGA are at increased risk of arterial stiffness and metabolic dysfunction.

## 1. Introduction

Low birth weight (LBW) is associated with increased risk of cardiovascular disease (CVD) and the related disorders, hypertension, stroke, and type-2 diabetes, later in life [1–3]. LBW may be due to preterm birth, poor fetal growth, or a combination of both. Although their relative importance is unknown, it is thought that intrauterine growth restriction (IUGR) is more important than preterm birth *per se* in the development of subsequent CVD [4]. With the increasing survival of extremely preterm infants, this may no longer be true. Early postnatal growth restriction, common in very preterm babies, may be as important in development of later organ dysfunction as IUGR at a similar gestational age (GA). Furthermore, such infants might have been exposed to glucocorticoids, either antenatally to accelerate lung maturation or postnatally to facilitate weaning from mechanical ventilation. This therapy may also result in later adverse renal or cardiovascular outcomes [5, 6]. 

Approximately 7% of babies are born preterm (<37 weeks gestation) [7] with 1% being with very LBW (<1500 g) or very preterm (<29 weeks gestation) [8]. To date, most long-term followup of very preterm infants have focused on neurodevelopmental and respiratory complications, with little attention to cardiovascular, renal, or metabolic outcomes. Suboptimal intrauterine nutrition may alter fetal programming during critical periods of growth, causing permanent changes in metabolism and cardiovascular or renal structure and function. If abnormal programming occurred postnatally in children born preterm, this would provide an opportunity for appropriate postnatal interventions. 

This study was undertaken to determine the relative influence of IUGR and preterm birth on subsequent cardiovascular, renal, and metabolic dysfunction. It was designed to be clinically relevant, practical, and easily achieved in settings with limited facilities.

## 2. Materials and Methods

### 2.1. Setting and Study Cohort

A retrospective cohort study was conducted at Royal North Shore Hospital (RNSH), Sydney, Australia. Children who had been born at RNSH between 1st January 1992 and 31st December 1995 with birth weight <1500 g or GA ≤32 weeks were identified from the neonatal intensive care records and studied at present time (2006–2008) to assess their cardiovascular, renal, and metabolic function. Recruitment of term subjects, who were born in 1992–95 and ≥37 weeks gestation, was obtained by advertisement in local media and through word-of-mouth by study participants. Subjects were divided into the following four groups based on gender-specific, birth weight percentiles by GA, Australian national data 1991–94 [9]: preterm and small for gestational age (Prem-SGA), preterm and appropriate for gestational age (Prem-AGA), term and SGA (Term-SGA), term and AGA (Term-AGA). Birth weights were converted to *z* score by the formula: (*x* − mean)/standard deviation.

Our sample size was calculated to detect a 10% difference in the primary outcomes with 80% power, for a two-tailed test and 5% significance level. For a 10% reduction in renal response following protein challenge in children with standard deviation of 5 mL/min [10], 25 were required in each group. For 10% systolic blood pressure (SBP) increase in 13-14-year-old children [11], 22 were required. We aimed to recruit 25 children into each of the four groups.

Ethics approval was granted by the Human Research Ethics Committee, Northern Sydney Central Coast, NSW Health as Protocol 0501-028M.

### 2.2. Outcome Measures

The primary outcomes were as follows.

- Cardiovascular function: SBP, diastolic blood pressure (DBP), and arterial augmentation index standardized at heart rate 75 bpm (AI at 75) [12] as a measure of arterial stiffness.
- Renal function: urine microscopy, urine protein/creatinine ratio, serum creatinine, and glomerular filtration rate (GFR) following oral protein load adjusted for body surface area (BSA), calculated as [urine creatinine concentration (mmol/L) × urine flow (mL/min) × 1.73]/[serum creatinine concentration (mmol/L) × BSA (m^2^)], as a measure of renal functional reserve (RFR).
- Metabolic function: fasting plasma glucose and serum insulin levels, homeostasis model of assessment for insulin resistance (HOMA-IR), and glucose and insulin levels following oral glucose load.

### 2.3. Procedure

Informed consent was obtained from both mothers and children. Children performed a self-assessment of puberty stage.

Subjects fasted overnight from 9 pm but were allowed plain water *ad libitum*. The first morning urine specimen was collected as midstream into boric acid, for urinary microscopy and measurement of protein/creatinine ratio. Upon arrival at 0850 hr, weight, height, and BP were measured. Body mass index (BMI) and BSA were calculated. BP was measured by standard mercury sphygmomanometry [13], and the average of duplicate readings 5 mins apart recorded. All clinical measurements were performed by one unblind investigator.

A second urine sample was tested for leucocytes, nitrites, protein, pH, blood, specific gravity, ketones, and glucose. Blood was collected for fasting glucose, insulin, creatinine, sodium, and potassium levels. 

An oral protein load 1 g/kg body weight [14], in the form of *Aussie Bodies* (Aussie Bodies Pty Ltd.)(drink: skim milk, Aussie Bodies Protein Blend [4.8%] (calcium caseinate, whey protein concentrate), fructose, skim milk powder, emulsifier (471), stabilisers (401, 407, 412), acidity regulator (331), flavours, colour (120), sweetener (955), contains milk, soy, and wheat. Bars: protein blend (31%) (isolated soy protein (tapioca starch, emulsifier (322), salt), whey protein isolate, calcium caseinate (soy lecithin)), milk chocolate compound (20%) (sugar, vegetable fat, milk solids, cocoa, cocoa mass, emulsifiers (492, 322), flavours, salt), glycerine, Aussie Bodies caramel (10%) (glucose, sweetened condensed milk, polydextrose, vegetable fat (antioxidant (307)), emulsifier (471), salt), glucose, peanuts (8%), cocoa (2%), emulsifier (322), vegetable fat, flavour) was taken over 30–45 mins. Oral glucose load 1.75 g/kg body weight to maximum 75 g [15] was taken over 10 mins (with *Glucaid*) after accounting for the glucose in *Aussie Bodies*. Subjects were given oral fluid loading 20 mL/kg body weight with water (after accounting for the fluid from protein and glucose drinks). 

Subjects reclined throughout the study period. The right radial pulse-wave characteristics were assessed by applanation tonometry (*SphygmoCor*, AtCor Medical, Australia) [16].

Urine samples were collected 1 and 2 hr after protein load. Volume and time were recorded, and urinary creatinine and glucose were measured. The amount voided was replaced with water orally. Further blood sample 2 hr after glucose load was collected for plasma glucose, serum insulin, and creatinine measurement.

### 2.4. Statistical Analyses

Analyses were performed using SPSS v16 for Windows (2007 SPSS Inc., Chicago). The Kruskal-Wallis test was used to compare continuous variables across the four groups, and chi-square test for proportions. Statistical tests were two tailed and statistically significant if *P* < .05.

To separate the relative influences of IUGR and preterm birth on cardiovascular, renal, and metabolic functions, multiple linear regression (MLR) analyses were performed for continuous outcomes. The study factors (size at birth and preterm birth) were categorized as either SGA or AGA and preterm (≤32 weeks gestation) or term (≥37 weeks gestation), to allow easier interpretation and clinical application. They were kept in the multivariate model, regardless of statistical significance. Other variables were included in the baseline multivariate model if the univariate *P* < .25. Variables not known to be confounders or nonsignificant (*P* ≥ .05) in the multivariate model were progressively eliminated, starting with the least significant.

Effect of modification on the two study factors was examined by including an interaction term in the model. Where the interaction term was significant (*P* < .05), further testing was done to determine which subgroups were significantly different from each other.

Assumptions of MLR in the final model were checked for normality, linearity, and homoscedasticity by examining residuals and normal probability plots. Collinearity problems were checked by stability of baseline models, parameter estimates, and standard errors.

## 3. Results

 A total of 71 periadolescent children were studied: 14 Prem-SGA (group 1), 25 Prem-AGA (group 2), 7 Term-SGA (group 3), and 25 Term-AGA (group 4). 

Table 1 summarizes the clinical characteristics and measurements, at birth and at the time of study. There were expected differences in GA, birth weight, and birth weight *z*-score amongst the four groups (*P* < .001). There were no significant differences in gender, age, weight, BMI, SBP, and DBP at time of study. The Prem-AGA group was taller than the other 3 groups at the time of study (*P* = .009), with an associated increased BSA.

Maternal characteristics of the children, at birth and time of study, are summarized (Table 2). The highest level of maternal educational attainment at the time of study is recorded; all mothers had at least secondary education. Of the Prem-SGA, 86% gave a history of preeclampsia during pregnancy compared with 16% Term-AGA (*P* < .001).

### 3.1. Cardiovascular Function

**Figure 1:**
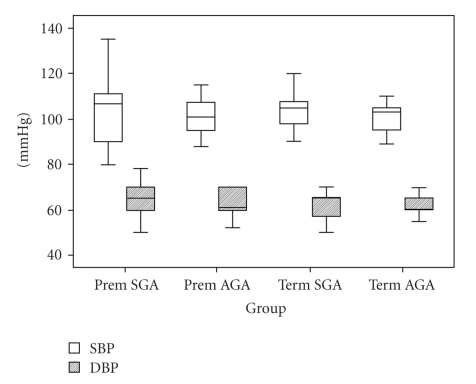
Box plot of systolic and diastolic blood pressure of children at the time of study.

SBP and DBP measurements are summarized in Figure 1. By *χ* ^2^ analysis, there were no significant differences in SBP or DBP across the 4 groups. However, in the multivariate analysis of SBP, SGA was a significant risk for increased SBP (*P* = .002), but preterm was not (*P* = .4) after adjusting for current BMI and DBP (Table 3). The model remained stable, without collinear problems, and interaction between the study factors was not significant (*P* = .53). SGA children had an average SBP 4.6 mmHg higher (95% CI: 1.8–7.3 mmHg) than those born AGA (Table 3).

**Figure 2:**
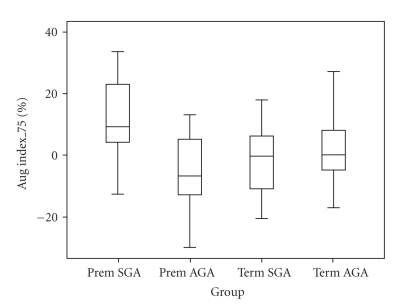
Box plot of augmentation index at heart rate 75 bpm of children at the time of study in 4 groups.

Arterial stiffness findings (AI at 75) are summarized in Figure 2. There were significant differences across the four groups (*χ* ^2^ = 12.21 with 3df, *P* = .007). In the multivariate analysis of AI at 75, BSA remained the only significant non-study variable in the model. There was a significant interaction between preterm and size for GA (*P* = .01). AI at 75 was significantly higher (average: 9.7%, 95% CI: 2.3–17.1%) in the subgroup Prem-SGA compared with all other groups (Table 4).

### 3.2. Renal Function

**Figure 3:**
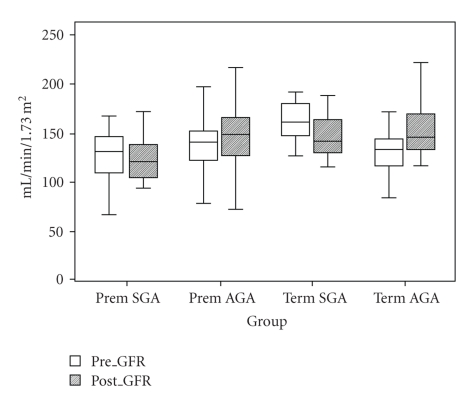
Box plot of glomerular filtration rate, corrected for body surface area before and after protein challenge of children in 4 groups.

Results for GFR (measured as true endogenous creatinine clearance) corrected for BSA, before and after protein load are summarized in Figure 3. There were no differences across the groups in GFR prior to protein load (*χ* ^2^ = 7.17 with 3df, *P* = .07), but results after protein loading showed a reduction in functional renal reserve in SGA children (*χ* ^2^ = 8.27 with 3df, *P* = .04). 

In the multivariate analysis of GFR after protein load, the only non-study factor variable remaining was GFR pre-protein (Table 5). Interaction between the study factors was not significant (*P* = .8). The GFR following protein load was significantly lower in SGA children (*P* = .01), while there was no difference in preterm children (*P* = .9). For a SGA child, the average GFR following protein load was 28.5 mL/min/1.73 m^2^ lower than that of an AGA child (95% CI: −50.1 to −6.8 mL/min/1.73 m^2^) (Table 5).

### 3.3. Metabolic Function

There was no significant difference across the four groups for HOMA-IR (*χ* ^2^ = 2.58 with 3df, *P* = .5), glucose level either at fasting (*χ* ^2^ = 2.81 with 3df, *P* = .4) or after glucose load (*χ* ^2^ = 5.72 with 3df, *P* = .13), and insulin level either at fasting (*χ* ^2^ = 2.36 with 3df, *P* = .5) or after glucose load (*χ* ^2^ = 1.77 with 3df, *P* = .6). 

In the multivariate analysis, neither being preterm (*P* = .4) nor SGA (*P* = .6) was significant for HOMA-IR. For 2-hr glucose level following glucose load, preterm birth was significant (*P* = .03) while being SGA was not (*P* = .9). In preterm children, the mean 2-hr glucose level (3.9 mmol/L) following glucose load was 0.25 mmol/L lower (95% CI: −0.48 to −0.02 mmol/L) than that of term children, a statistically but not clinically significant difference. However, being both preterm and SGA was significantly associated with increased 2-hr insulin level following glucose load; the average level (35.6 mIU/L) was 15.5 mIU/L higher (95% CI: 5.6–25.5 mIU/L, *P* = .003) compared with all other groups (Table 6).

## 4. Discussion

In this cohort of periadolescent children, we have shown subtle but definite and different long-term effects of both IUGR and preterm. These effects were evident in cardiovascular, renal, and metabolic function.

### 4.1. Cardiovascular Function: Blood Pressure

Our result on the risk of increased SBP in children born with IUGR is consistent with some [3, 17, 18] but not all [19–21] previous studies. In some of the latter publications, it is not possible to separate the effects of preterm and IUGR, because of noncomparability of their study groups [19, 20]. Johansson et al. [21] reported an increased risk of high SBP in SGA born preterm amongst young adult men. These results are compatible with ours. BP is “tracked” across age [22], and any effect of low birth weight on BP will be magnified in an older cohort study [23]. In addition, young adult men are generally at greater risk of cardiovascular disease than young women [24]. Changes could be more subtle in our subjects because they are younger. Huxley et al. [25] suggested that the inverse relationship between birth weight and SBP may be spurious, because of adjustment for current weight by investigators. However, a more recent meta-analysis by Gamborg et al. [17] concluded that the relationship was present regardless of current BMI—this is consistent with our results. Although the average difference in SBP between SGA and AGA groups (3.7 mmHg) was reduced without adjustment for current BMI (4.6 mmHg), it was still significant (*P* = .02).

The subjects were 12–15 years old when this study was conducted. Adolescents experience complex physiological changes which may confound/mask an association between birth weight and SBP [26]. Despite this, we found an inverse association between birth weight and SBP, consistent with other studies [27, 28].

### 4.2. Cardiovascular Function: Arterial Stiffness (AI at 75)

Our finding of increased arterial stiffness in children born both preterm and SGA is consistent with the findings of Cheung et al. [29] and Singhal et al. [30]. Several studies have failed to distinguish between low birth weight related to preterm and that due specifically to IUGR [31–33]. This makes comparisons to our findings impossible. A recent systematic review [34] concluded that poor fetal growth and preterm birth produce different patterns of altered vascular system development, with different implications for cardiovascular health in adult life.

The mechanism whereby discordance between birth weight and GA leads to an increase in arterial stiffness in preterm children remains unclear. The reported impairment of endothelial function in preterm and SGA individuals [30] suggests functional alteration of arterial tone contributing to the increase in systemic arterial stiffness. While the underlying cause is yet to be revealed, whether epigenetic modification [35], accelerated telomere ablation [36], or other mechanisms, it appears that altered vascular physiology associated with LBW is largely irreversible. Children born SGA and/or very preterm should therefore be followed up for future cardiovascular risk.

### 4.3. Renal Function: Renal Functional Reserve (RFR)

As we did not expect major overt renal functional impairment, GFR under protein-loaded conditions was measured to detect subtle changes in RFR. It is clear from our results that IUGR was associated with reduced RFR (*P* = .01) while preterm *per se *was not (*P* = .9). This may lead to earlier appearance of the reduced GFR encountered in disorders such as hypertension, type-2 diabetes, and the metabolic syndrome. These findings are consistent with previous studies describing associations of LBW with severity of expression and progression of kidney disease from various causes [37–39] and indicate real value for early screening in these individuals. 

Long-term data on renal function in subjects born very preterm are few. Two studies compared renal function in preterm subjects or those with IUGR with full-term subjects [40, 41]. In the first, reduced creatinine clearances were found in 40 school-age children born preterm compared with 43 controls of similar age (but no differences were detected when Preterm-SGA and AGA groups were compared with each other) [40]. In the second, examining young adult women, the Term-SGA and preterm groups showed a nonsignificant reduction in GFR, but no Preterm-SGA subjects were included [41]. Neither study measured the effect of protein loading on renal function and therefore did not assess RFR.

A recent study [42] investigating 23 Preterm-SGA, 29 Preterm-AGA (<32 weeks), and 30 Term-AGA controls, at a mean age of 20.7 years showed that Preterm-SGA subjects had a lower baseline GFR (using inulin clearance) than either Preterm-AGA or control. Unfortunately, there was no Term-SGA group included, making it difficult to separate the effects of IUGR and preterm. Stimulation by high-protein meals increased GFR in all three groups. Surprisingly (given that baseline GFR was lower in the Preterm-SGA group), following adjustment for current BSA, stimulated GFR was not significantly different among the groups. 

Brenner and colleagues [43, 44] hypothesized that IUGR may cause a low nephron number, thus predisposing to later hypertension and renal disease. Subsequent studies showed that LBW and IUGR were associated with mild to moderate elevations of BP [45], reduced numbers of compensatory hypertrophied glomeruli [46–48], lower GFR, and higher urine albumin-to-creatinine ratio [37, 49]. Preterm infants have fewer glomeruli than infants born at term [50]. Therefore, both IUGR and preterm may lead to reduced number of nephrons.

Although preterm birth was not found to be an independent risk factor for renal dysfunction in our study, larger studies should be undertaken and adults with preterm births followed-up as renal function decreases with age.

### 4.4. Metabolic Function

Using HOMA-IR [51] as an outcome measure of insulin resistance, neither being born preterm (*P* = .4) nor SGA (*P* = .6) was significant risk factor. Similarly, fasting glucose and fasting insulin levels were not significantly different across the four groups. 

This finding is consistent with other studies [30, 52] which found fasting glucose and insulin levels comparable between Preterm-SGA and AGA children [52].

With glucose levels 2 hr after glucose load as outcome measure, being preterm was significant (*P* = .03) while being SGA was not (*P* = .9). In preterm children, the mean 2-hr glucose level was 0.25 mmol/L lower (95% CI −0.48 to −.02 mmol/L) than that in term children. Although statistically significant, such a difference is not clinically significant. This is in contrast to Hovi et al. [53] who reported significantly higher 2 hr glucose concentrations in preterm LBW young adults compared with term controls. The difference in findings could be due to an older age group studied (range: 18–27 years), in contrast to our study mean age of 13.8 years (range: 11.3–15.6 years). Similar to our study, the differences between SGA and AGA in the preterm groups were not significant. 

In children born both preterm and SGA, the average 2-hr insulin level following glucose load was 15.5 mIU/L higher (95% CI: 5.6–25.5 mIU/L) than in all other groups. This suggests that poor fetal growth and preterm birth are both significant risk factors for diabetes in later life. The results support the previously reported inverse association between birth weight and later development of diabetes [2, 54–58] and provide evidence for an interaction between preterm and poor fetal growth. Hofman et al. [59] found that children born preterm, whether AGA or SGA, had reduced insulin sensitivity similar to that seen in term-SGA. Hovi et al. [53] also reported that young adults born preterm with very LBW (<1500 g) had higher 2 hr glucose concentration and more insulin resistance than those born at term. A recent study has suggested that the association between LBW and risk for diabetes is mediated through poor fetal growth and preterm birth [60]. Another study has shown an inverse relationship between birth weight and fasting plasma glucose, following glucose load and hemoglobin A1C [61], but information regarding GA and preterm birth was not given. A recent systematic review concluded that birth weight was inversely related to a risk of type-2 diabetes [62].

## 5. General Discussion

The findings presented here support the hypothesis that children born with IUGR are at risk of later systolic hypertension, cardiovascular, renal, and metabolic dysfunction. Among children born preterm, only those with IUGR appear to be at increased risk of arterial stiffness and metabolic dysfunction. It has been recognised for over 30 years that maternal undernutrition during pregnancy results in infants of LBW and that these infants exhibit in late adult life an increased risk of cardiovascular morbidity, renal disease and type II diabetes mellitus [63], all associated with an increased rate of preterm death. More recent publications have shown that the increase in these disorders is already present in young adults and is found to be not only consequent on maternal under-nutrition but also more specifically on fetal growth restriction from a variety of causes [64, 65]. In the current study, evidence is presented that subtle but significant differences in cardiovascular function, renal function, and glucose metabolism are detectable in LBW infants even before adult life. In addition, it appears that preterm birth and IUGR expose infants to separate and probably additive risks of these adverse outcomes.

The limitations of this study must be acknowledged. The main problem encountered was recruiting children for a study involving venepuncture. Recruitment of children is more difficult and challenging compared with adults [66, 67]. The period of recruitment was limited, to reduce the potential confounding impact of developments in neonatal care. Some of the nonsignificant results may be due to the sample size being too small to detect differences. Pre-eclampsia is frequently associated with IUGR. It is also a significant risk factor for renal dysfunction and associated with metabolic syndrome—larger numbers may expose a relationship. Nevertheless, several significant results despite the sample size suggest that the differences are relatively large. 

As puberty affects cardiovascular, renal, and metabolic functions, another limitation of the study is that pubertal stage was not formally assessed. As there were no significant age differences across the groups, and age was further tested as a potential confounder in regression analyses, one would expect the effects of puberty to be equal across all groups despite the variable onset of puberty. Ideally, children would be followed after puberty, as young adults.

The findings of this study indicate pressing reasons for supervision of the health and nutrition of all LBW infants—at least into adult life—regardless of the cause of their LBW. Since the advent of specialised neonatal intensive care, the survival of extremely preterm and/or growth-restricted babies has increased significantly. Efforts to identify individuals at increased disease risk well before any clinical manifestation provide a window of time that may allow delay or prevention of overt disease. Clearly, screening of this population for abnormalities will be most achievable if the measurements are clinically and technically simple, relatively noninvasive, and inexpensive. All of the measurements made in this study meet these criteria. They can be performed in a community setting and require only basic diagnostic laboratory facilities. 

That such significant findings were evident in a relatively small cohort study provides strong support for a larger community-based examination of blood pressure, arterial stiffness, renal functional reserve, and glucose tolerance. The relevant preventive measures and interventions which can be achieved postnatally will need to be assessed and evaluated in further well-designed studies.

## Figures and Tables

**Table 1:** Clinical characteristics and measurements of children at birth and at the time of study in 4 groups.

	Prem-SGA *N* = 14	Prem-AGA *N* = 25	Term-SGA *N* = 7	Term-AGA *N* = 25	*P* value*
At Birth					

Gender					
Female (%)	7 (50%)	11 (44%)	2 (29%)	12 (48%)	.8
Gestational age (wk)					
Median	31	30	39	40	<.001
IQR	28.8–31.0	27.5–31.0	38.0–40.0	38.5–41.0	
Birth weight (g)					
Median	980	1635	2750	3302	<.001
IQR	768–1038	991–1850	2430–2870	3105–3690	
Birth weight *z* score					
Median	−1.59	0.30	−1.58	−0.30	<.001
IQR	−2.20–1.49	−0.27–0.85	−2.35–1.41	−0.62–0.33	

Time of Study					

Age (yr)					
Median	13.5	14.1	13.6	13.6	.06
IQR	12.48–13.97	13.66–15.03	12.35–14.83	12.54–14.78	
Weight (kg)					
Median	44.3	56.0	51.2	47.2	.06
IQR	31.63–52.13	45.50–60.15	40.00–56.50	41.60–53.25	
Height (cm)					
Median	153.9	164.8	156.9	156.6	.009
IQR	146.00–160.98	157.25–169.65	149.50–167.00	151.65–162.55	
BMI (kg/m^2^)					
Median	17.74	19.74	19.03	18.27	.4
IQR	15.33–21.06	17.98–21.27	17.45–21.80	17.38–20.42	
BSA (m^2^)					
Median	1.40	1.62	1.52	1.46	.03
IQR	1.15–1.49	1.43–1.72	1.29–1.59	1.36–1.54	
SBP (mmHg)					
Median	107	101	105	103	.6
IQR	90.0–114.5	95.0–107.5	95.0–110.0	103.0–105.0	
DBP (mmHg)					
Median	65	61	65	60	.7
IQR	58.0–70.5	60.0–70.0	55.0–66.0	60.0–65.0	

Abbreviations: BSA: body surface area; BMI: body mass index; DBP: diastolic blood pressure; IQR: interquartile range; SBP: systolic blood pressure; Prem-AGA: preterm appropriate for gestational age; Prem-SGA: preterm small for gestational age; Term-AGA: term appropriate for gestational age; Term-SGA: term small for gestational age.

*For comparisons across 4 groups, the Kruskal-Wallis test was used for continuous variables and the Pearson chi-square test for discrete variables.

**Table 2:** Maternal characteristics of children at birth and at the time of study in 4 groups.

	Prem-SGA *N* = 14	Prem-AGA *N* = 25	Term-SGA *N* = 7	Term-AGA *N* = 25	*P* value**
Preeclampsia in index pregnancy*	12 (86%)	3 (12%)	2 (29%)	4 (16%)	<.001
Maternal gestational diabetes*	0	0	0	1 (4%)	.6
Maternal education^#^					
Secondary	5 (36%)	9 (36%)	2 (29%)	1 (4%)	.04
Tertiary	9 (64%)	16 (64%)	5 (71%)	24 (96%)	
Maternal smoking^#^	1 (7%)	2 (8%)	1 (14%)	1 (4%)	.8

*At Birth.

^#^At the Time of Study.

**For comparisons between 4 groups, chi-square tests were used.

**Table 3:** Coefficients for dependent variable: systolic blood pressure.

Model	Coeff.	SE	*t*	*P*	95% CI	
					Lower	Upper
(Constant)	9.72	7.14	1.36	.178	−4.55	23.98
Preterm*	−1.01	1.27	−0.79	.431	−3.55	1.53
Birth Size*	4.57	1.39	3.30	.002	1.80	7.34
Current BMI^#^	1.11	0.24	4.69	<.001	0.64	1.58
DBP^#^	1.12	0.11	10.42	<.001	0.90	1.33

*Binary. ^#^Continuous.

**Table 4:** Coefficients of augmentation index at heart rate 75 bpm for subgroup Prem-SGA.

Model	Coeff	SE	*t*	*P*	95% CI	
					Lower	Upper
(Constant)	36.82	11.25	3.27	.002	14.36	59.27
Prem_SGA	9.70	3.70	2.62	.011	2.31	17.09
BSA (DuBois)*	−25.78	7.39	−3.49	.001	−40.53	−11.02

*Body Surface area (using Du Bois method).

**Table 5:** Coefficients for dependent variable: glomerular filtration rate following protein load, corrected for body surface area.

Model	Coeff	SE	*t*	*P*	95% CI	
					Lower	Upper
(Constant)	63.14	23.65	2.67	.010	15.92	110.35
Preterm	−0.79	9.83	−0.08	.936	−20.40	18.825
Birth Size	−28.48	10.84	−2.63	.011	−50.12	−6.835
GFR1Corr*	0.68	0.17	4.07	<.001	0.35	1.01

*GFR before protein load.

**Table 6:** Coefficients for dependent variable: insulin level after 2 hrs glucose challenge for Prem-SGA.

Model	Coeff	SE	*t*	*P*	95% CI	
					Lower	Upper
(Constant)	−573.81	155.74	−3.69	<.001	−884.92	−262.69
Prem_SGA	15.541	5.00	3.11	.003	5.56	25.53
Ht	3.86	1.01	3.81	<.001	1.84	5.88
Wt	−6.54	1.69	−3.87	<.001	−9.92	−3.16
Current BMI	17.08	4.20	4.07	<.001	8.69	25.46
F_Insulin*	1.84	0.42	4.40	<.001	1.00	2.67
Mat_Ed**	−11.93	4.38	−2.73	.008	−20.67	−3.18

*Fasting Insulin.

**Maternal Education (2 categories: secondary, tertiary).

## References

[1] Rich-Edwards JW, Stampfer MJ, Manson JE (1997). Birth weight and risk of cardiovascular disease in a cohort of women followed up since 1976. *British Medical Journal*.

[2] Lithell HO, McKeigue PM, Berglund L, Mohsen R, Lithell U-B, Leon DA (1996). Relation of size at birth to non-insulin dependent diabetes and insulin concentrations in men aged 50–60 years. *British Medical Journal*.

[3] Law CM, De Swiet M, Osmond C (1993). Initiation of hypertension in utero and its amplification throughout life. *British Medical Journal*.

[4] Barker DJP, Osmond C, Simmonds SJ, Wield GA (1993). The relation of small head circumference and thinness at birth to death from cardiovascular disease in adult life. *British Medical Journal*.

[5] Bensky AS, Kothadia JM, Covitz W (1996). Cardiac effects of dexamethasone in very low birth weight infants. *Pediatrics*.

[6] Wintour EM, Moritz KM, Johnson K, Ricardo S, Samuel CS, Dodic M (2003). Reduced nephron number in adult sheep, hypertensive as a result of prenatal glucocorticoid treatment. *Journal of Physiology*.

[7] Roberts CL, Algert CS, Peat B, Henderson-Smart DJ (2004). Trends in place of birth for preterm infants in New South Wales, 1992–2001. *Journal of Paediatrics and Child Health*.

[8] Tucker J, McGuire W (2004). Epidemiology of preterm birth. *British Medical Journal*.

[9] Roberts CL, Lancaster PAL (1999). Australian national birthweight percentiles by gestational age. *Medical Journal of Australia*.

[10] De Santo NG, Capasso G, Anastasio P (1990). The renal hemodynamic response following a meat meal in children with chronic renal failure and in healthy controls. *Nephron*.

[11] National High Blood Pressure Education Program Working Group on Hypertension Control in Children and Adolescents (2004). The fourth report on the diagnosis, evaluation, and treatment of high blood pressure in children and adolescents. *Pediatrics*.

[12] Wilkinson IB, MacCallum H, Flint L, Cockcroft JR, Newby DE, Webb DJ (2000). The influence of heart rate on augmentation index and central arterial pressure in humans. *Journal of Physiology*.

[13] Perloff D, Grim C, Flack J (1993). Human blood pressure: determination by sphygmomanometry. *Circulation*.

[14] Hellerstein S, Berenbom M, Erwin P, Wilson N, DiMaggio S (2004). Measurement of renal functional reserve in children. *Pediatric Nephrology*.

[15] Alberti KG, Zimmet PZ (1998). Definition, diagnosis and classification of diabetes mellitus and its complications—part 1: diagnosis and classification of diabetes mellitus. Provisional report of a WHO consultation. *Diabetic Medicine*.

[16] Smith SA, Morris JM, Gallery EDM (2004). Methods of assessment of the arterial pulse wave in normal human pregnancy. *American Journal of Obstetrics and Gynecology*.

[17] Gamborg M, Byberg L, Rasmussen F (2007). Birth weight and systolic blood pressure in adolescence and adulthood: meta-regression analysis of sex- and age-specific results from 20 nordic studies. *American Journal of Epidemiology*.

[18] Bonamy A-KE, Norman M, Kaijser M (2008). Being born too small, too early, or both: does it matter for risk of hypertension in the elderly?. *American Journal of Hypertension*.

[19] Irving RJ, Belton NR, Elton RA, Walker BR (2000). Adult cardiovascular risk factors in premature babies. *Lancet*.

[20] Bonamy A-KE, Bendito A, Martin H, Andolf E, Sedin G, Norman M (2005). Preterm birth contributes to increased vascular resistance and higher blood pressure in adolescent girls. *Pediatric Research*.

[21] Johansson S, Iliadou A, Bergvall N, Tuvemo T, Norman M, Cnattingius S (2005). Risk of high blood pressure among young men increases with the degree of immaturity at birth. *Circulation*.

[22] Chen X, Wang Y (2008). Tracking of blood pressure from childhood to adulthood: a systematic review and meta-regression analysis. *Circulation*.

[23] Davies AA, Smith GD, May MT, Ben-Shlomo Y (2006). Association between birth weight and blood pressure is robust, amplifies with age, and may be underestimated. *Hypertension*.

[24] Reckelhoff JF (2001). Gender differences in the regulation of blood pressure. *Hypertension*.

[25] Huxley R, Neil A, Collins R (2002). Unravelling the fetal origins hypothesis: is there really an inverse association between birthweight and subsequent blood pressure?. *Lancet*.

[26] Federico G, Baroncelli GI, Vanacore T, Fiore L, Saggese G (2003). Pubertal changes in biochemical markers of growth. *Hormone Research*.

[27] Leon DA, Johansson M, Rasmussen F (2000). Gestational age and growth rate of fetal mass are inversely associated with systolic blood pressure in young adults: an epidemiologic study of 165,136 Swedish men aged 18 years. *American Journal of Epidemiology*.

[28] Adair LS, Cole TJ (2003). Rapid child growth raises blood pressure in adolescent boys who were thin at birth. *Hypertension*.

[29] Cheung YF, Wong KY, Lam BCC, Tsoi NS (2004). Relation of arterial stiffness with gestational age and birth weight. *Archives of Disease in Childhood*.

[30] Singhal A, Kattenhorn M, Cole TJ, Deanfield J, Lucas A (2001). Preterm birth, vascular function, and risk factors for atherosclerosis. *Lancet*.

[31] Tauzin L, Rossi P, Giusano B (2006). Characteristics of arterial stiffness in very low birth weight premature infants. *Pediatric Research*.

[32] Leeson CPM, Kattenhorn M, Morley R, Lucas A, Deanfield JE (2001). Impact of low birth weight and cardiovascular risk factors on endothelial function in early adult life. *Circulation*.

[33] Lurbe E, Torro MI, Carvajal E, Alvarez V, Redón J (2003). Birth weight impacts on wave reflections in children and adolescents. *Hypertension*.

[34] Norman M (2008). Low birth weight and the developing vascular tree: a systematic review. *Acta Paediatrica*.

[35] Martin H, Lindblad B, Norman M (2007). Endothelial function in newborn infants is related to folate levels and birth weight. *Pediatrics*.

[36] Fuster JJ, Díez J, Andrés V (2007). Telomere dysfunction in hypertension. *Journal of Hypertension*.

[37] Hoy WE, Rees M, Kile E, Mathews JD, Wang Z (1999). A new dimension to the Barker hypothesis: low birthweight and susceptibility to renal disease. *Kidney International*.

[38] Al Salmi I, Hoy WE, Kondalsamy-Chennakes S, Wang Z, Healy H, Shaw JE (2008). Birth weight and stages of CKD: a case-control study in an Australian population. *American Journal of Kidney Diseases*.

[39] White SL, Perkovic V, Cass A (2009). Is low birth weight an antecedent of CKD in Later Life? A systematic review of observational studies. *American Journal of Kidney Diseases*.

[40] Rodríguez-Soriano J, Aguirre M, Oliveros R, Vallo A (2005). Long-term renal follow-up of extremely low birth weight infants. *Pediatric Nephrology*.

[41] Kistner A, Celsi G, Vanpee M, Jacobson SH (2000). Increased blood pressure but normal renal function in adult women born preterm. *Pediatric Nephrology*.

[42] Keijzer-Veen MG, Kleinveld HA, Lequin MH (2007). Renal function and size at young adult age after intrauterine growth restriction and very premature birth. *American Journal of Kidney Diseases*.

[43] Brenner BM, Garcia DL, Anderson S (1988). Glomeruli and blood pressure. Less of one, more the other?. *American Journal of Hypertension*.

[44] Mackenzie HS, Lawler EV, Brenner BM (1996). Congenital oligonephropathy: the fetal flaw in essential hypertension?. *Kidney International*.

[45] Eriksson J, Forsén T, Tuomilehto J, Osmond C, Barker D (2000). Fetal and childhood growth and hypertension in adult life. *Hypertension*.

[46] Manalich R, Reyes L, Herrera M, Melendi C, Fundora I (2000). Relationship between weight at birth and the number and size of renal glomeruli in humans: a histomorphometric study. *Kidney International*.

[47] Hughson M, Farris AB, Douglas-Denton R, Hoy WE, Bertram JF (2003). Glomerular number and size in autopsy kidneys: the relationship to birth weight. *Kidney International*.

[48] Hughson MD, Douglas-Denton R, Bertram JF, Hoy WE (2006). Hypertension, glomerular number, and birth weight in African Americans and white subjects in the southeastern United States. *Kidney International*.

[49] Keijzer-Veen MG, Schrevel M, Finken MJJ (2005). Microalbuminuria and lower glomerular filtration rate at young adult age in subjects born very premature and after intrauterine growth retardation. *Journal of the American Society of Nephrology*.

[50] Rodríguez MM, Gómez AH, Abitbol CL, Chandar JJ, Duara S, Zilleruelo GE (2004). Histomorphometric analysis of postnatal glomerulogenesis in extremely preterm infants. *Pediatric and Developmental Pathology*.

[51] Matthews DR, Hosker JP, Rudenski AS (1985). Homeostasis model assessment: insulin resistance and *β*-cell function from fasting plasma glucose and insulin concentrations in man. *Diabetologia*.

[52] Willemsen RH, De Kort SWK, Van Der Kaay DCM, Hokken-Koelega ACS (2008). Independent effects of prematurity on metabolic and cardiovascular risk factors in short small-for-gestational-age children. *Journal of Clinical Endocrinology and Metabolism*.

[53] Hovi P, Andersson S, Eriksson JG (2007). Glucose regulation in young adults with very low birth weight. *New England Journal of Medicine*.

[54] Rich-Edwards JW, Colditz GA, Stampfer MJ (1999). Birthweight and the risk for type 2 diabetes mellitus in adult women. *Annals of Internal Medicine*.

[55] Carlsson S, Persson P-G, Alvarsson M (1999). Low birth weight, family history of diabetes, and glucose intolerance in Swedish middle-aged men. *Diabetes Care*.

[56] Eriksson JG, Forsén T, Tuomilehto J, Osmond C, Barker DJP (2003). Early adiposity rebound in childhood and risk of Type 2 diabetes in adult life. *Diabetologia*.

[57] Curhan GC, Willett WC, Rimm EB, Spiegelman D, Ascherio AL, Stampfer MJ (1996). Birth weight and adult hypertension, diabetes mellitus, and obesity in US men. *Circulation*.

[58] Forsen T, Eriksson J, Tuomilehto J, Reunanen A, Osmond C, Barker D (2000). The fetal and childhood growth of persons who develop type 2 diabetes. *Annals of Internal Medicine*.

[59] Hofman PL, Regan F, Jackson WE (2004). Premature birth and later insulin resistance. *New England Journal of Medicine*.

[60] Kaijser M, Bonamy A-KE, Akre O (2009). Perinatal risk factors for diabetes in later life. *Diabetes*.

[61] Al Salmi I, Hoy WE, Kondalsamy-Chennakesavan S (2008). Disorders of glucose regulation in adults and birth weight: results from the Australian diabetes, obesity and lifestyle (AusDiab) study. *Diabetes Care*.

[62] Whincup PH, Kaye SJ, Owen CG (2008). Birth weight and risk of type 2 diabetes a systematic review. *Journal of the American Medical Association*.

[63] Ravelli GP, Stein ZA, Susser MW (1976). Obesity in young men after famine exposure in utero and early infancy. *New England Journal of Medicine*.

[64] Barker DJP (1997). Fetal nutrition and cardiovascular disease in later life. *British Medical Bulletin*.

[65] Barker DJP (2007). The origins of the developmental origins theory. *Journal of Internal Medicine*.

[66] Collet JP, Floret D, Cochat P (1991). Group meetings for recruitment of children in a clinical trial. *Therapie*.

[67] Caldwell PH, Murphy SB, Butow PN, Craig JC (2004). Clinical trials in children. *Lancet*.

